# A numerical framework for an electrically-charged PCM brick to reduce winter peak heating demand

**DOI:** 10.1038/s41598-025-29854-x

**Published:** 2025-12-11

**Authors:** Riyadh Alturki, Ali B. M. Ali, Omar J. Alkhatib, Ibrahim Mahariq

**Affiliations:** 1 Civil Engineering Department, College of Engineering, Imam Mohammad Ibn Saud Islamic University (IMSIU), 11432 Riyadh, Saudi Arabia; 2 Advanced Technical College, University of Warith Al-Anbiyaa, Karbala, Iraq; 3 Architectural Engineering Department, College of Engineering, UAE University, Al Ain, United Arab Emirates; 4 University College, Korea University, Seoul, 02481 South Korea; 5 Department of Medical Research, China Medical University Hospital, China Medical University, Taichung, Taiwan

**Keywords:** Phase change material (PCM), Thermal energy storage, Building envelope, Peak load shaving, Active heating, Cold climate, Energy science and technology, Engineering

## Abstract

The substantial peak electrical demand for space heating in cold and freezing climates poses a significant challenge to grid stability and energy affordability. This study proposes and numerically investigates a novel active thermal energy storage system integrated directly into a building brick to address this challenge. The system features an encapsulated Phase Change Material (PCM) composite, enhanced with a high-conductivity copper oxide foam, and is coupled with a low-wattage electrical heating element. This design enables the brick to function as a ‘thermal battery,’ charging with off-peak electricity and discharging heat during peak demand periods. A comprehensive computational fluid dynamics (CFD) model was developed to analyze the system’s performance under severe winter conditions, with ambient temperatures as low as − 30 °C and varying electrical power inputs. The results demonstrate a profound improvement in the indoor thermal environment. While an unheated brick’s surface dropped to − 5 °C, the active system maintained it above a stable + 8 °C, delivering a peak heat output of over 150 W/m² to the living space. This effective load shifting reduced the wall’s net daily energy loss by nearly 70%, significantly lessening the burden on the primary HVAC system during peak hours. The findings confirm that the proposed active PCM-brick is a highly effective and viable solution for peak-shaving, enhancing occupant comfort, and improving the energy resilience of buildings in cold climates.

## Introduction

The global imperative to decarbonize the energy sector has placed significant pressure on buildings, which account for approximately 40% of total energy consumption and over a third of energy-related CO₂ emissions worldwide^1–4^. In regions characterized by cold or freezing climates, the vast majority of this energy is dedicated to space heating, which often represents the single largest load on residential and commercial utility grids^5–7^. The reliance on conventional heating systems during peak demand periods—typically early mornings and evenings—imposes immense strain on electrical infrastructure, necessitating the use of inefficient and carbon-intensive “peaker” power plants and contributing to grid instability^7–9^. Furthermore, the increasing electrification of the heating sector, driven by the adoption of heat pumps and electric resistance heaters as alternatives to fossil fuels, is projected to exacerbate these peak-load challenges^10–12^. Consequently, developing innovative and intelligent building envelope technologies that can manage thermal energy more effectively is not merely an objective for enhancing comfort, but a critical strategy for improving grid stability, reducing energy costs, and facilitating a sustainable energy transition^13–15^.

A highly promising approach for addressing these challenges lies in the application of Thermal Energy Storage (TES) systems within the building fabric itself^16–21^. TES technologies enable the capture and storage of energy during periods of low demand or low cost (e.g., overnight) and its subsequent release during periods of high demand, a process known as load shifting or peak-shaving^22–25^. Among various TES methods, Latent Heat Thermal Energy Storage (LHTES) using Phase Change Materials (PCMs) has garnered significant research interest for building applications^26–29^. PCMs are substances that can absorb and release large quantities of thermal energy at a nearly constant temperature during their phase transition (typically solid-to-liquid and vice-versa). This characteristic offers a distinct advantage over sensible heat storage materials, providing a much higher energy storage density within a narrow temperature range, which is ideal for maintaining occupant comfort^28,30–33^. However, a major practical challenge associated with many PCMs, particularly organic variants, is their inherently low thermal conductivity. This property can severely limit the rate of heat transfer, leading to slow and inefficient charging and discharging cycles unless a thermal conductivity enhancer is integrated^34–36^.

The integration of PCMs into building envelopes has been extensively investigated as a passive strategy, primarily for cooling applications in moderate to hot climates^37–41^. Researchers have successfully incorporated PCMs into various building components, including wallboards^42–45^, concrete^46–48^, plaster, and by impregnating them into the cavities of hollow bricks^49–51^. In these passive systems, the PCM absorbs solar and ambient heat during the day to prevent overheating and releases it at night. While effective for reducing cooling loads, the applicability of such passive strategies in cold or freezing climates during the heating season is severely limited. In these environments, passive solar gains are often insufficient, unreliable, or ill-timed to fully “charge” (i.e., melt) the PCM, rendering the latent heat capacity of the material largely inaccessible when it is most needed^43,52–55^.

To unlock the full potential of PCMs for heating applications in cold regions, an active charging strategy is required. This involves coupling the PCM-integrated building component with a controllable energy source. Electrical resistance heating presents an ideal candidate for this purpose due to its simplicity, low capital cost, and perfect compatibility with off-peak electricity tariffs^56–59^. By integrating a low-wattage electrical heating element with a PCM-enhanced building envelope, it becomes possible to create a "thermal battery." During off-peak hours at night, when electricity is abundant and inexpensive, the heater can be activated to charge the PCM. This stored thermal energy can then be passively discharged throughout the subsequent day to meet the building’s heating requirements, thereby drastically reducing or even eliminating the need to draw expensive electricity from the grid during peak hours^60–65^. This concept of an electrically-charged thermal mass aligns perfectly with the goals of demand-side management and the development of grid-interactive efficient buildings^66–68^.

Despite the clear conceptual advantages, the design and implementation of an active, electrically-coupled PCM heating system within a standard building component like a brick presents unique and complex challenges^69,70^. The system must be designed to ensure uniform and efficient charging of the PCM, effective heat release to the indoor space, and long-term material stability, all while operating within a harsh freezing environment. While some studies have explored electrically-coupled PCM storage for underfloor heating or standalone storage units, research on a fully integrated, brick-based wall heating and storage system remains scarce.

This study, therefore, aims to address this critical research gap by proposing and numerically investigating a novel building component: a brick integrated with an encapsulated PCM and coupled with an electrical wall heating element, specifically designed for peak-load reduction in freezing climates. The novelty of this work lies in the synergistic combination of these technologies to create an active thermal battery within the building envelope itself. By using a comprehensive numerical model, this research will analyze the system’s dynamic thermal behavior, its effectiveness in shifting electrical loads, and its overall impact on reducing peak energy consumption. The findings are intended to provide fundamental insights and design guidelines for a new generation of smart building envelopes capable of actively interacting with the electrical grid, thereby enhancing energy resilience, reducing heating costs, and supporting the broader integration of renewable energy sources.

## Methodology and numerical formulation

This section outlines the comprehensive framework established to simulate and analyze the proposed active thermal energy storage system. The discussion begins by defining the physical problem and its operational context, followed by the governing mathematical models and boundary conditions. The numerical simulations were performed using a proprietary in-house computational fluid dynamics (CFD) code, which has been developed based on the Finite Volume Method (FVM). The solver is specifically engineered to handle the complex physics of the current problem, including conjugate heat transfer and solid–liquid phase transitions via the enthalpy-porosity formulation. The fidelity of this in-house code is demonstrated through a rigorous validation study presented in "Validation study" section. Finally, it details the rigorous verification and validation procedures undertaken to ensure the accuracy and reliability of the numerical results.

### Problem description

**Fig. 1:**
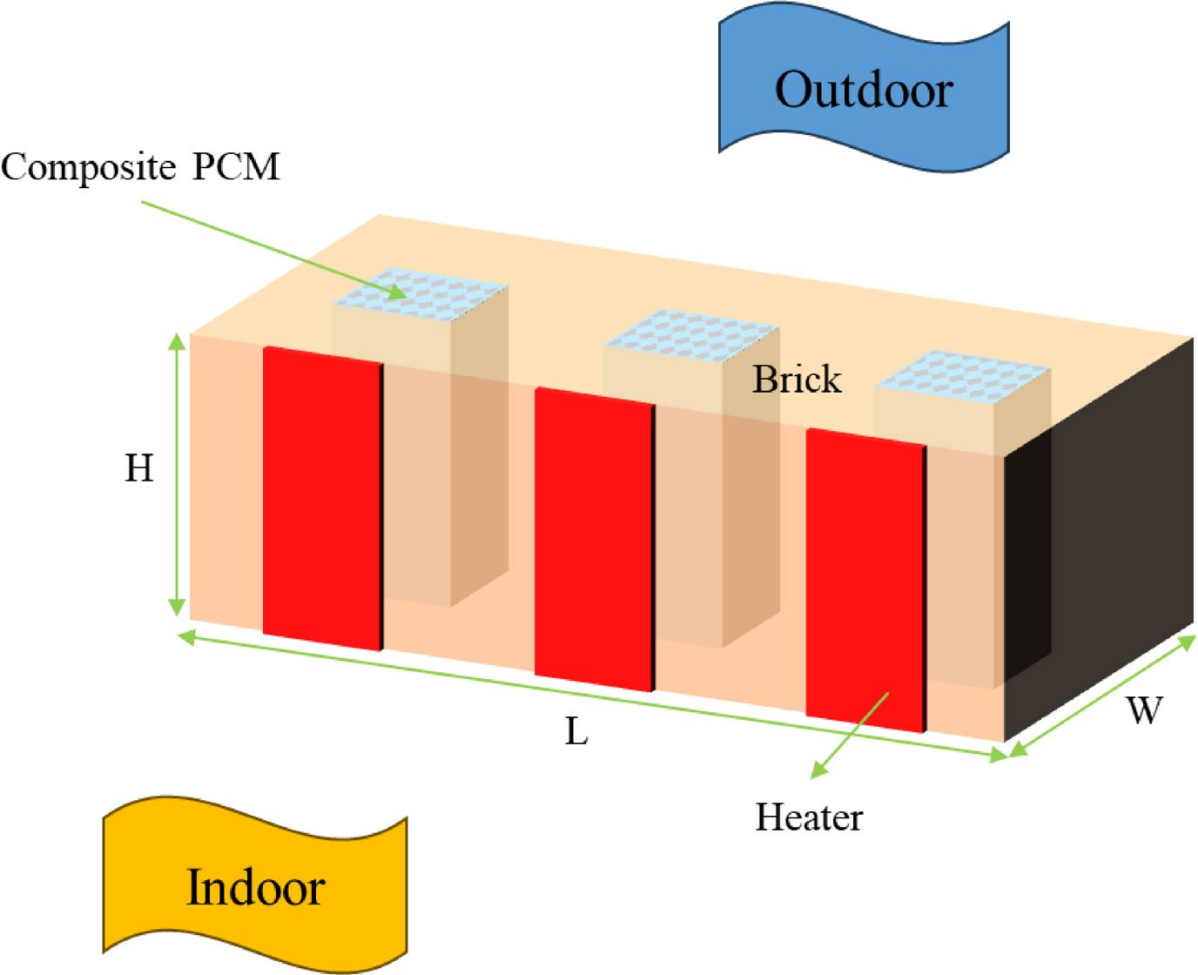
Schematic cross-section of the active PCM-brick, illustrating its internal components, material composition, and the thermal boundary conditions applied in the numerical model.

This study addresses the critical challenge of high peak electrical demand for space heating in buildings situated in cold and freezing climates. The proposed solution is a novel “thermal battery” brick, an active building envelope component designed specifically for electrical load shifting. The physical system consists of a standard clay brick integrated with a central 4 cm × 4 cm square enclosure (Fig. 1). This cavity houses a composite material comprising Pentadecane, a low-temperature Phase Change Material (PCM), and a high-conductivity Copper Oxide Foam (COF). The COF is characterized by a high porosity of 0.95 and a fine pore diameter of 0.1 mm, serving not only as a structural matrix but, more critically, as a thermal conductivity enhancer to accelerate the otherwise slow thermal response of the organic PCM. The selection of Pentadecane as the PCM is critical and was based on its thermophysical properties, particularly its melting temperature (T_m_) of 10 °C and narrow melting temperature range (T_mr_) of 1 °C. The melting temperature was strategically chosen to strike a balance between efficient charging and effective heat discharge. A melting point of 10 °C allows the PCM to act as an effective thermal buffer, releasing stored latent heat to maintain the indoor brick surface at a stable and comfortable temperature (e.g., above 8 °C, as shown in our results) even when the external temperature is well below freezing. This temperature is sufficiently high to provide useful radiant heat to the indoor environment (maintained at 20 °C) but low enough to be fully and efficiently charged using a low-power heating element during off-peak periods. Furthermore, the narrow melting range is highly desirable as it ensures that the phase transition occurs at a near-constant temperature, providing stable and predictable thermal regulation, which is essential for both occupant comfort and reliable peak-shaving performance

.

It is important to note the strategic placement of the electrical heating element on the indoor surface of the brick rather than in direct contact with the PCM. This configuration was chosen for three primary reasons. First, it provides a crucial dual function: during the off-peak charging period at night, the heat that is not immediately absorbed by the brick provides useful baseline heating to the indoor space, fulfilling the building’s simultaneous heating demand. Second, the brick material acts as a thermal diffuser, promoting more uniform heating of the PCM core and preventing localized overheating and potential degradation of the PCM. Finally, this surface-mounted design is significantly simpler and more cost-effective from a manufacturing and long-term durability perspective compared to embedding a heater within the sealed PCM composite.

The central innovation of this system is the integration of a low-wattage electrical heating element, which provides a uniform power output (P). To assess the system’s robustness and performance under realistic and challenging conditions, this study parametrically investigates the effect of varying this power input. The operational strategy is to leverage this heater to actively charge the PCM by melting it and storing latent heat during off-peak electricity periods, typically overnight. This stored thermal energy is then passively discharged into the indoor space during the subsequent peak demand hours. This process is designed to maintain indoor thermal comfort while substantially reducing or eliminating the need to draw expensive power from the grid.

### Mathematical formulation

To capture the complex physics of the system, which involves conjugate heat transfer, buoyancy-driven fluid flow, and solid–liquid phase transition, a robust mathematical model is essential. The formulation is based on the well-established framework presented by Nassr et al. (2025), which has been successfully applied to similar PCM systems. The accuracy of the simulation is fundamentally dependent on the thermophysical properties (TPP) of the constituent materials, which are detailed in Table 1.

**Table 1:** Thermophysical properties of the materials used in the simulation^49^.

TPP	Brick	Pentadecane	COF
$\rho$	1800	768	6500
${C}_{p}$	829	2070	530
k	0.72	0.15	20
$\beta$	–	9.45 *${10}^{-4}$	–
$\mu$	–	2.87 *${10}^{-3}$	–
${T}_{m}$	–	10	–
${T}_{mr}$	–	1	–
h	–	208	–

The fluid flow of the molten Pentadecane is modeled as incompressible, requiring the satisfaction of the continuity equation to ensure mass conservation within any given control volume^49^.

1 $$\frac{\partial u}{{\partial x}} + \frac{\partial v}{{\partial y}} + \frac{\partial w}{{\partial z}} = 0$$

Momentum transport within the liquid PCM is described by the Navier–Stokes equations (Eqs. 2–4). A key feature of this model is the inclusion of the Boussinesq approximation, (ρβ)ₚcₘ(T – Tₘ)g, which accounts for the natural convection currents driven by temperature-induced density gradients. To elegantly handle the solid–liquid phase transition without explicitly tracking the interface, the enthalpy-porosity formulation is employed. This technique introduces a momentum sink term that is a function of the liquid fraction (λ), effectively immobilizing the fluid in solid regions while having no effect in fully liquid regions^49^.

2 $$\rho_{PCM} \left( {\frac{\partial u}{{\partial t}} + u\frac{\partial u}{{\partial x}} + v\frac{\partial u}{{\partial y}} + w\frac{\partial u}{{\partial z}}} \right) = - \frac{\partial p}{{\partial x}} + \mu_{PCM} \left( {\frac{{\partial^{2} u}}{{\partial x^{2} }} + \frac{{\partial^{2} u}}{{\partial y^{2} }} + \frac{{\partial^{2} u}}{{\partial z^{2} }}} \right) - a\frac{{(1 - \lambda )^{2} }}{{\lambda^{3} + b}}u$$

3 $$\rho_{PCM} \left( {\frac{\partial v}{{\partial t}} + u\frac{\partial v}{{\partial x}} + v\frac{\partial v}{{\partial y}} + w\frac{\partial v}{{\partial z}}} \right) = - \frac{\partial p}{{\partial x}} + \mu_{PCM} \left( {\frac{{\partial^{2} v}}{{\partial x^{2} }} + \frac{{\partial^{2} v}}{{\partial y^{2} }} + \frac{{\partial^{2} v}}{{\partial z^{2} }}} \right) - a\frac{{(1 - \lambda )^{2} }}{{\lambda^{3} + b}}v$$

4 $$\rho_{PCM} \left( {\frac{\partial w}{{\partial t}} + u\frac{\partial w}{{\partial x}} + v\frac{\partial w}{{\partial y}} + w\frac{\partial w}{{\partial z}}} \right) = - \frac{\partial p}{{\partial z}} + \mu_{PCM} \left( {\frac{{\partial^{2} w}}{{\partial x^{2} }} + \frac{{\partial^{2} w}}{{\partial y^{2} }} + \frac{{\partial^{2} w}}{{\partial z^{2} }}} \right) - (\rho \beta )_{PCM} (T - T_{s} )g - a\frac{{(1 - \lambda )^{2} }}{{\lambda^{3} + b}}w$$

Heat transfer throughout the system is governed by a set of energy equations. For the complex PCM region where both convection and phase change occur, a single enthalpy-based energy equation is used (Eq. 5), which inherently accounts for both sensible and latent heat effects. In the solid domains of the brick and COF, this formulation simplifies to the standard transient heat conduction equation^49^.

5 $$\rho_{PCM} \left( {C_{p,PCM} + h_{PCM} \frac{d\lambda }{{dT}}} \right)\frac{\partial T}{{\partial t}} + (\rho C_{p} )_{PCM} \left( {u\frac{\partial T}{{\partial x}} + v\frac{\partial T}{{\partial y}} + w\frac{\partial T}{{\partial z}}} \right) = k_{PCM} \left( {\frac{{\partial^{2} T}}{{\partial x^{2} }} + \frac{{\partial^{2} T}}{{\partial y^{2} }} + \frac{{\partial^{2} T}}{{\partial z^{2} }}} \right)$$

6 $$(\rho_{b} C_{p,b} )\frac{{\partial T_{b} }}{\partial t} = k_{b} \left( {\frac{{\partial^{2} T_{b} }}{{\partial x^{2} }} + \frac{{\partial^{2} T_{b} }}{{\partial y^{2} }} + \frac{{\partial^{2} T_{b} }}{{\partial z^{2} }}} \right)$$

7 $$(\rho_{b} C_{p,a} )\frac{{\partial T_{a} }}{\partial t} = k_{a} \left( {\frac{{\partial^{2} T_{a} }}{{\partial x^{2} }} + \frac{{\partial^{2} T_{a} }}{{\partial y^{2} }} + \frac{{\partial^{2} T_{a} }}{{\partial z^{2} }}} \right)$$

To track the progress of the phase transition, a liquid fraction parameter, λ, is defined. A smooth error function (erf) is chosen to describe this transition (Eq. 8), as it provides a numerically stable and physically representative progression from solid (λ = 0) to liquid (λ = 1) over the defined melting temperature range (T_mr_)^49^.

8 $$\lambda = 0.5\,erf\left( {4\frac{{T_{s} - T_{m} }}{{T_{mr} }}} \right) + 0.5$$

### Boundary and initial conditions

To solve the governing equations, a set of well-defined boundary conditions that reflect the system’s real-world operating environment is required. The indoor surface of the brick, exposed to the conditioned living space, is modeled with a convective boundary condition where the temperature is maintained at T_indoor_ = 20 °C via a heat transfer coefficient of h_indoor_ = 20 W/m K. The outdoor surface is subjected to dynamic winter conditions. To model this, the 24-h ambient temperature profile was simulated as a sinusoidal function with a daily mean temperature set to Tmin + 5 °C and an amplitude of 5 °C, ensuring the temperature cycle reaches the prescribed minimum (T_min_) during the early morning hours. The minimum ambient temperatures T_min_ tested in this study range down to an extreme of -30 °C; these scenarios were intentionally chosen not to represent a single specific city, but to conduct a parametric stress-test of the technology’s effectiveness and operational limits under a wide variety of demanding cold climate conditions. The heat transfer to the outdoor environment is governed by a combined convective and radiative heat transfer coefficient of h_os_ = 25 W/m^2^ K. This surface is also exposed to active thermal loads with a defined daily schedule: daytime solar radiation of q′′ = 700 W/m^2^ is applied for 8 h (from t = 08:00 to t = 16:00), and the integrated heating element is activated during a prescribed 8-h off-peak charging period at night (from t = 22:00 to t = 06:00). The power input (P) for the heating element is parametrically analyzed at 0, 100, 200, and 300 W/m^2^. To accurately represent the thermal behavior of a single brick within an extensive wall assembly, the top, bottom, and side faces of the brick are treated as adiabatic (zero-gradient) boundaries. The simulation for each case begins with the entire domain at a uniform initial temperature corresponding to the ambient condition at t = 0.

### Mesh and time-step independence

Ensuring the numerical results are free from discretization errors is a critical step in model verification. To this end, rigorous independence studies were conducted for a representative case (Tₘᵢₙ =  − 20 °C). The simulation was performed with progressively finer meshes and smaller time-steps, with the peak heating power saved during the discharge cycle serving as the key monitoring variable. The results of this analysis, presented in Table 2 and Table 3, indicate that a computational mesh of approximately 410,000 cells and a time-step of 5 s provide a convergent solution. Since further refinement of either the mesh or the time-step yielded negligible changes in the outcome (< 0.2%), these settings were adopted for all subsequent simulations to ensure an optimal balance of numerical accuracy and computational efficiency.

**Table 2:** Mesh independence study results.

Mesh level	Number of cells	Peak heating power saved (W/m^2^)	Relative difference (%)
Coarse	220,000	45.81	–
Medium	410,000	46.53	1.55
Fine	850,000	46.59	0.13

**Table 3:** Time-step independence study results.

Time-step (s)	Peak heating power saved (W/m^2^)	Relative difference (%)
15	46.88	–
10	46.61	− 0.58
5	46.53	− 0.17

### Validation study

**Fig. 2:**
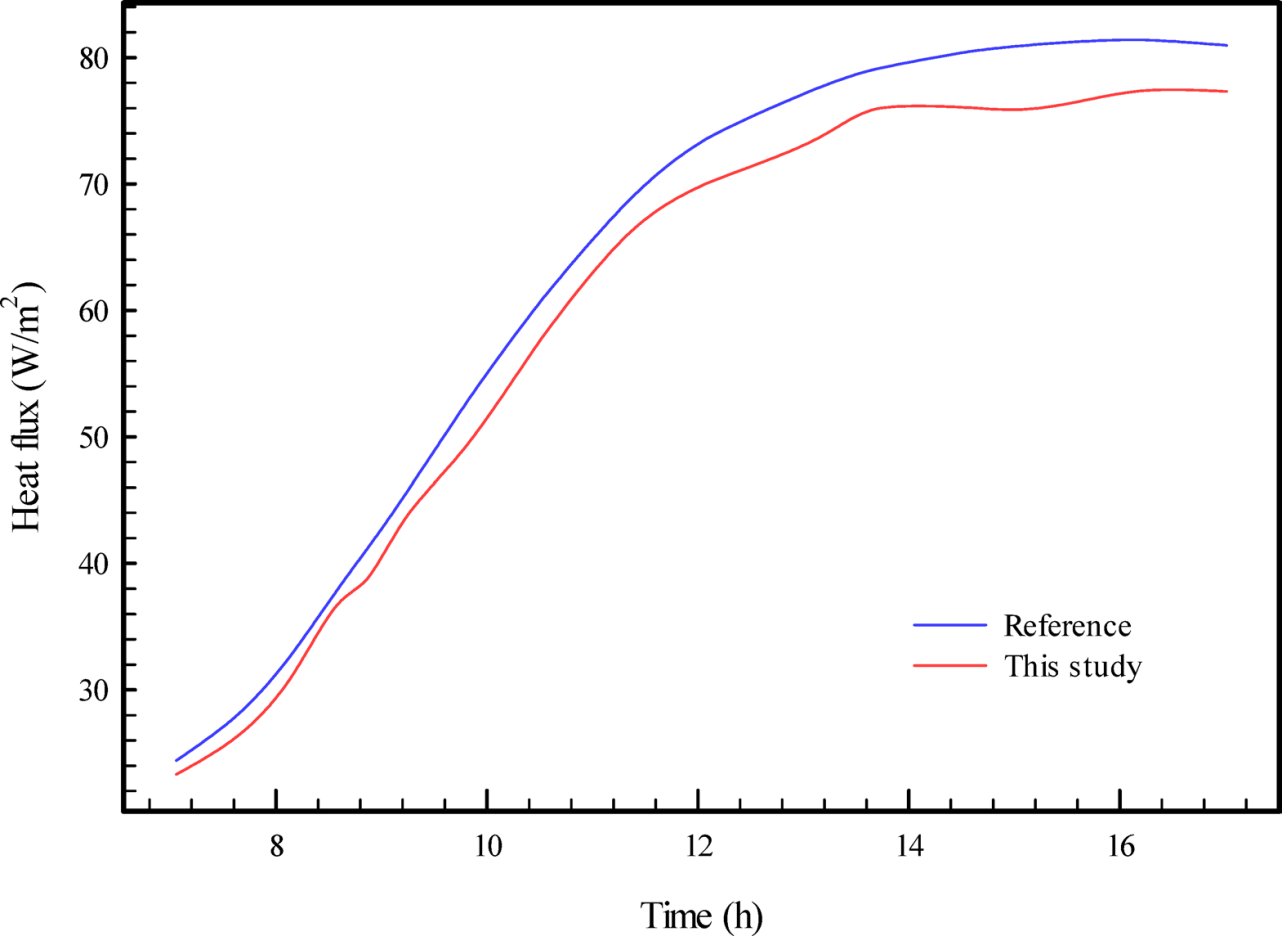
Result of validation study^3^.

To benchmark the performance of the in-house numerical solver and ensure its fidelity, a validation study was conducted against the established findings of Alawadhi (2008)^3^. For this comparative analysis, a specific configuration from the reference paper was meticulously recreated, focusing on the case where the PCM-filled cylinders were positioned nearer the interior environment (Hc = H/3). The temporal profile of the indoor surface heat flux, as predicted by our simulation, is plotted alongside the reference data in Fig. 2. A high degree of congruence is observed between the two curves throughout the daily cycle, with a mean quantitative discrepancy of approximately 5%. This successful benchmark lends significant credibility to the simulation framework’s ability to accurately capture the complex thermal dynamics of PCM-integrated building components and substantiates the validity of the results presented in this work.

### Performance metrics

To quantify the overall daily energy impact of the active brick on the building’s heating load, the Daily Peak Demand Contribution (Pdc) was calculated. This metric represents the net thermal energy per unit area that the primary HVAC system must supply over a 24-h period to compensate for the heat transfer through the brick wall. It is calculated by numerically integrating the instantaneous indoor surface heat flux (q′′_is_) over a full 24-h cycle (T = 86,400 s), as shown in the equation below:

9 $${\text{Pdc }}\left( {{\text{kWh}}/{\text{m}}^{{2}} } \right) \, = \, - \, \left( {{1 }/{ 3},{6}00,000} \right) \, * \, \int {{\text{o}}^{{\text{T}}} } {\text{ q}}_{{{\text{is}}}}^{\prime \prime } \left( {\text{t}} \right){\text{ dt}}$$

Here, q′′_is_ is given in W/m^2^. The negative sign is applied so that a net heat loss from the indoor space over the day (a negative integral) results in a positive Pdc value, which intuitively represents a required energy input. The constant factor of 1/3,600,000 is used to convert the final result from Joules/m^2^ to the more conventional unit of kWh/m^2^.

## Results and discussion

This section presents a detailed analysis of the numerical simulation results. The discussion is structured to evaluate the performance of the active PCM-brick system by first examining its direct impact on the indoor thermal environment, followed by an analysis of the energy dynamics at the building envelope, and concluding with a visualization of the internal heat storage mechanism.

### Indoor thermal environment and comfort

**Fig. 3:**
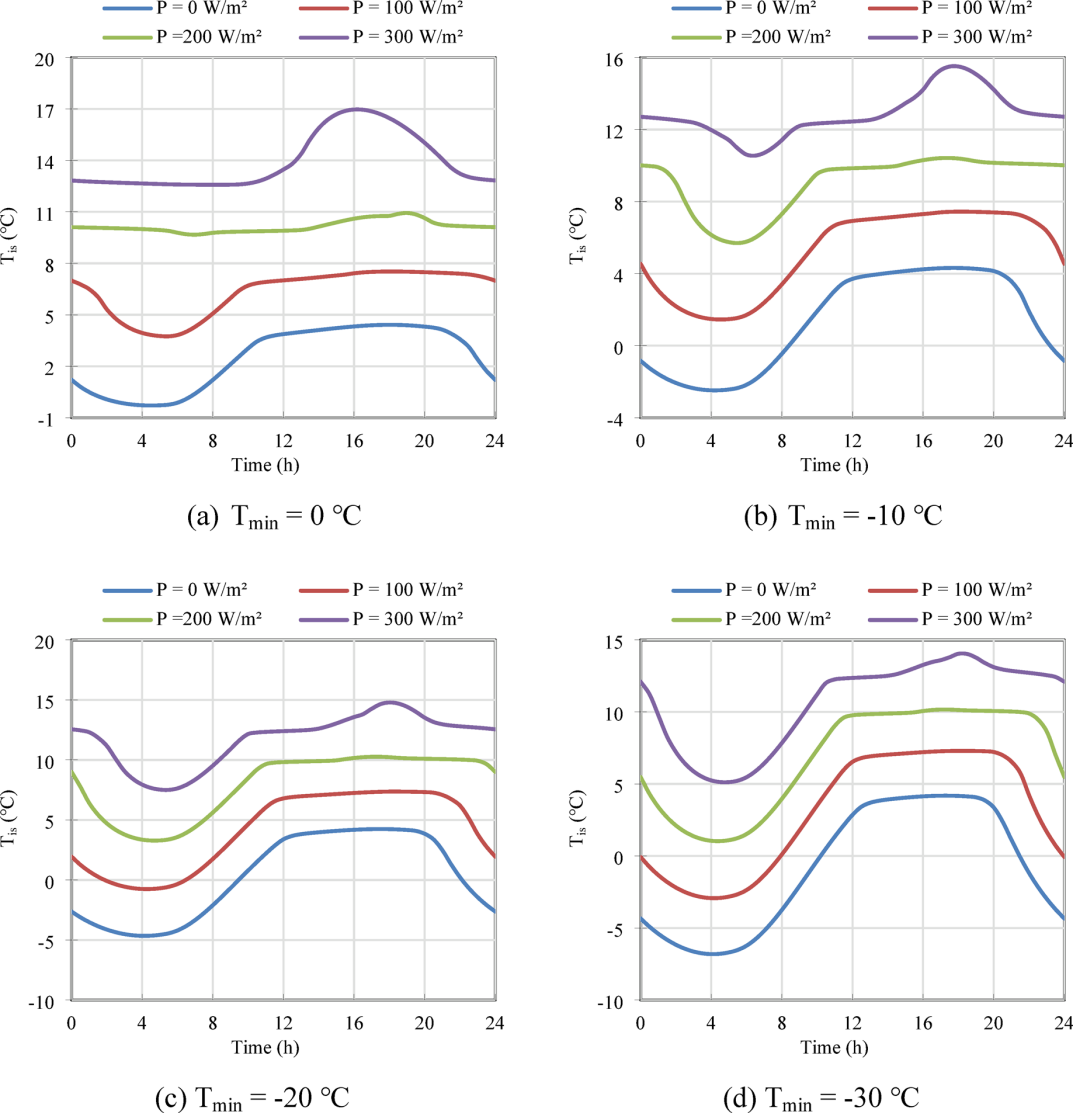
Temporal variation of the indoor surface temperature (T_is_) for different integrated heating powers (P) under four minimum ambient temperature scenarios: (**a**) T_min_ = 0 °C, (**b**) Tₘᵢₙ =  − 10 °C, (**c**) Tₘᵢₙ =  − 20 °C, and (**d**) T_min_ = -30 °C.

The primary function of the proposed active brick is to transform a typically cold exterior wall into a stable, warm surface, thereby enhancing occupant comfort and providing useful heat. The impact of the integrated heating system on this critical metric is illustrated in Fig. 3, which displays the temporal variation of the indoor surface temperature (T_is_) over a 24-h cycle for four different freezing conditions.

In the baseline case with no heating (P = 0 W/m^2^), the brick acts as a significant thermal liability. As the outdoor temperature drops, the indoor surface temperature plummets, falling well below a comfortable level. For instance, in the Tₘᵢₙ =  − 20 °C scenario (Fig. 3c), the unheated wall’s surface temperature drops to a minimum of approximately − 5 °C, creating an extremely cold radiant surface that would drastically increase the building’s overall heating load and cause significant occupant discomfort.

The application of electrical heating fundamentally alters this behavior. Even a modest power input of 100 W begins to stabilize the temperature, while higher powers (200 W/m^2^ and 300 W/m^2^) transform the wall into an effective low-temperature radiant heating panel. Quantitatively, for the Tₘᵢₙ =  − 20 °C case, applying 200 W/m^2^ of power elevates the minimum indoor surface temperature from − 5 °C to a much more manageable + 5 °C. At 300 W/m^2^, the temperature is maintained above 8 °C throughout the entire day. A crucial qualitative observation is the flattening of the temperature profile at higher power inputs. This stabilization is a direct result of the Phase Change Material’s activation. The electrical energy input during the off-peak charging period is absorbed as latent heat by the melting Pentadecane. This stored energy is then released as the PCM solidifies, creating a thermal buffer that dampens the effect of the fluctuating outdoor temperature and provides a more consistent heat output to the room.

### Peak load reduction and heat flux dynamics

**Fig. 4:**
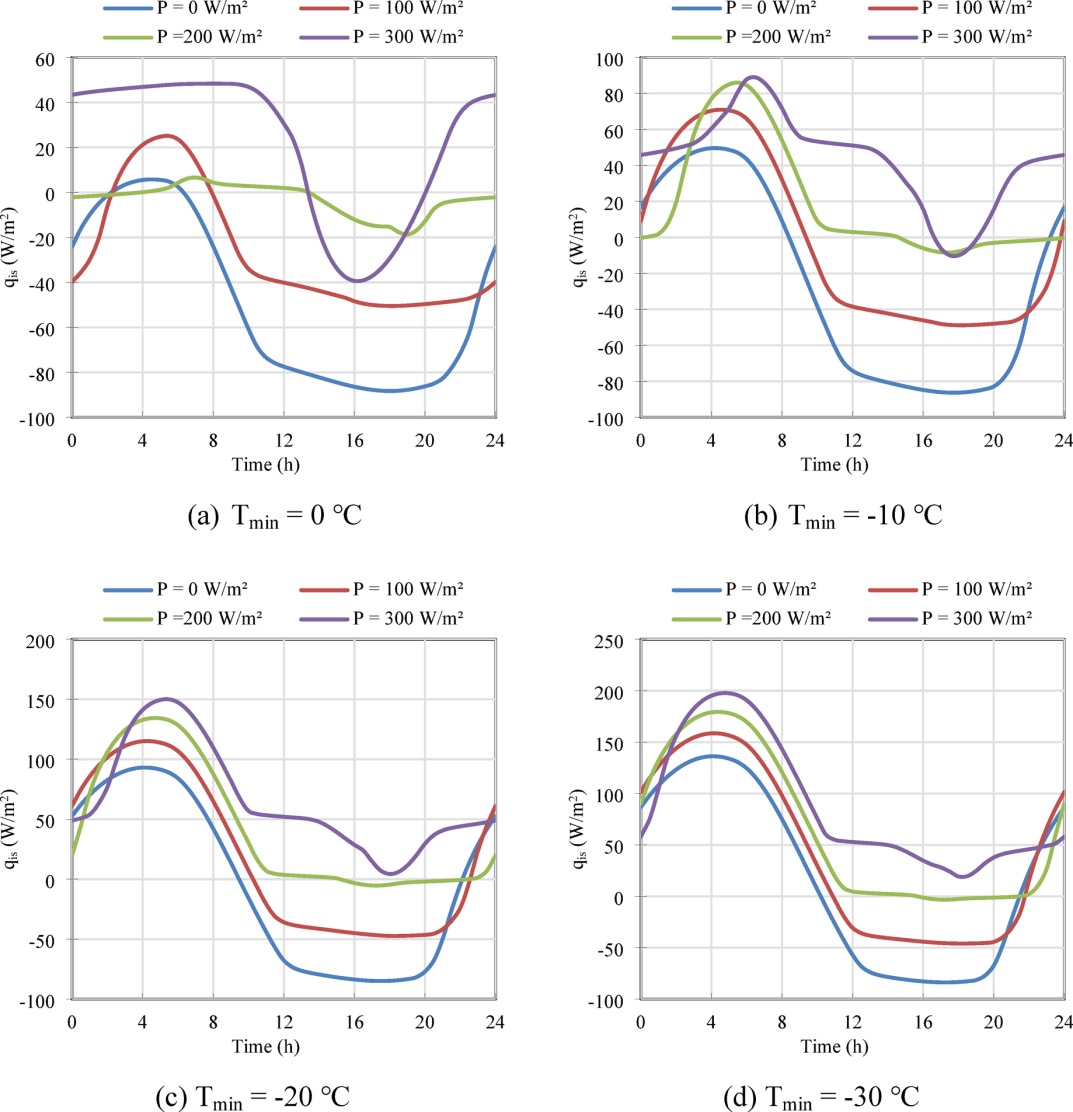
The 24-h profile of the indoor surface heat flux (q′′_is_) for different heating powers (P) under four minimum ambient temperature scenarios: (**a**) T_min_ = 0 °C, (**b**) Tₘᵢₙ =  − 10 °C, (**c**) T_min_ =  − 20 °C, and (**d**) Tₘᵢₙ =  − 30 °C.

The effectiveness of the system in shifting electrical loads is best understood by analyzing the heat flux at the indoor surface (q′′ᵢₛ), as shown in Fig. 4. Here, positive values represent a heat gain for the indoor space (the wall is heating the room), while negative values indicate heat loss.

The P = 0 W/m^2^ case again serves as the benchmark, demonstrating a continuous and significant heat loss from the building. At Tₘᵢₙ =  − 20 °C (Fig. 4c), the peak heat loss reaches nearly -50 W/m^2^, representing a constant drain on the building’s primary heating system. When the integrated heater is activated, the direction of the heat flux is reversed. The wall transitions from an energy sink to an energy source. The stored thermal energy is discharged into the room, particularly during the coldest hours of the day.

This behavior is the core of the peak-shaving functionality. For the Tₘᵢₙ =  − 20 °C case, the 300 W/m^2^ system delivers a peak heat output of over 150 W/m^2^ to the indoor space. This is thermal energy that was generated and stored using off-peak electricity, and is now being delivered during a time that would typically coincide with the building’s peak heating demand. By providing this supplemental heat, the active brick directly reduces the load that must be met by the primary, peak-hour heating system. The sinusoidal shape of the heat output is a result of the dynamic balance between the stored energy being released and the continuous heat loss to the freezing outdoor environment. As the outdoor temperature reaches its minimum (typically in the early morning), the temperature gradient across the brick increases, driving a higher rate of heat discharge to the indoor space.

### Impact on daily energy demand

**Fig. 5:**
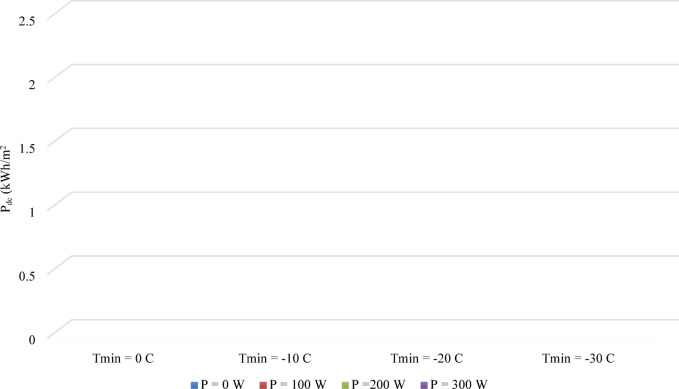
The effect of integrated heating power (P) and minimum ambient temperature (T_min_) on the net daily heat loss per unit area (Pdc) from the indoor environment.

To quantify the overall effectiveness of the system, the net daily energy impact is considered. Figure 5 presents the daily peak demand contribution (Pdc), which represents the net heat loss from the wall that must be compensated by the primary HVAC system over a 24-h period.

The results clearly show that as the outdoor environment becomes colder, the net daily heat loss for the unheated brick (P = 0 W) increases significantly, from approximately 1.2 kWh/m^2^ at T_min_= 0 °C to over 2.0 kWh/m^2^ at Tₘᵢₙ =  − 30 °C. The application of the integrated heating system systematically reduces this net loss. This metric effectively demonstrates how much of the wall’s inherent heat loss is offset by the stored and discharged energy. For instance, at Tₘᵢₙ =  − 20 °C, applying 300 W/m^2^ of heating power reduces the daily compensatory energy requirement from around 1.6 kWh/m^2^ to just 0.5 kWh/m^2^. This represents a reduction of nearly 70% in the energy that the primary heating system must supply to overcome the thermal losses through this section of the building envelope.

An important and counter-intuitive trend is observable in Fig. 5 for the higher heating power inputs (P = 200 and 300 W/m^2^), where the net daily heat loss (Pdc) is lower for the T_min_ =  − 20 °C scenario than for T_min_ =  − 10 °C. This phenomenon highlights an operational “sweet spot” for the thermal battery. At − 10 °C, the outdoor temperature is not cold enough to drive a complete discharge of the PCM’s stored latent heat during the day, meaning some of the stored off-peak energy goes unused. In contrast, the T_min_ =  − 20 °C condition provides a sufficient thermal gradient to force a more complete daily discharge cycle, maximizing the effective use of the stored energy to offset daytime heat loss. However, at the extreme T_min_ =  − 30 °C condition, the overall heat loss through the envelope becomes too great, overwhelming the system’s storage capacity and causing the Pdc to increase once more.

### Internal thermal storage visualization

**Fig. 6:**
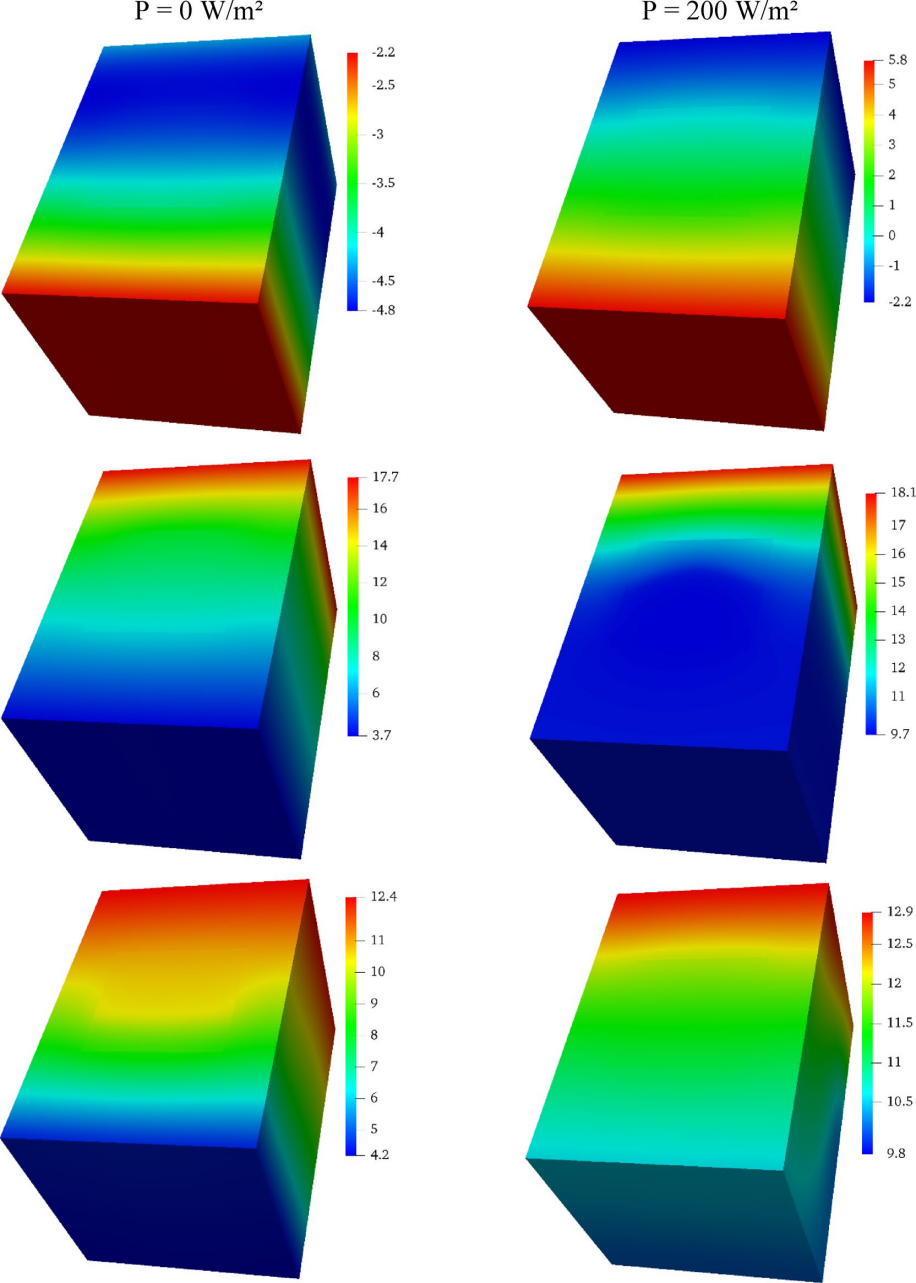

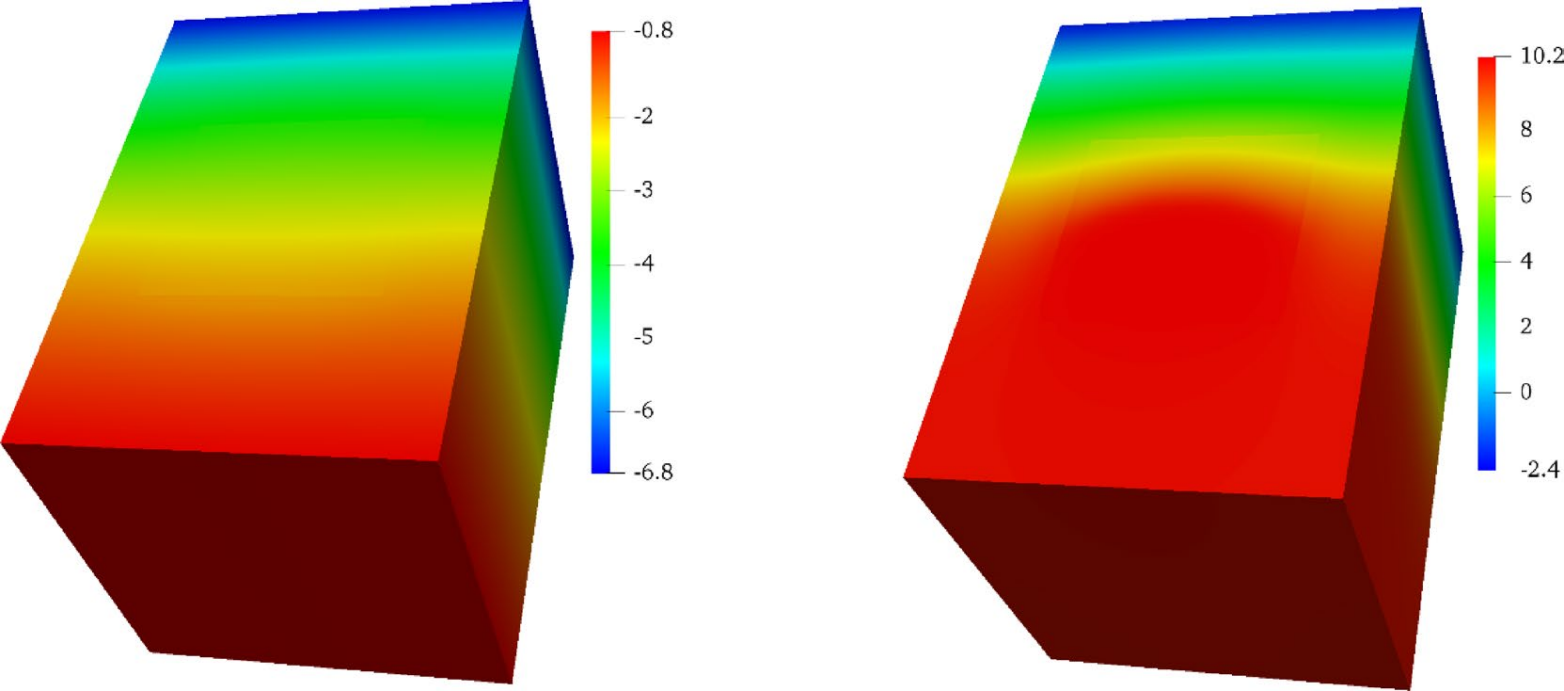
Temperature distribution contours within the PCM-brick for the Tₘᵢₙ =  − 10 °C scenario. The left column shows the unheated case (P = 0 W/m^2^), while the right column shows the heated case (P = 200 W/m^2^) at corresponding times of day (from top to bottom: early charging, peak charge, and discharging phase).

To provide a deeper physical insight into the heat storage and transfer mechanisms, Fig. 6 displays the temperature distribution contours within the brick’s cross-section at various times for the Tₘᵢₙ =  − 10 °C scenario.

The contours for the unheated brick (left column) reveal a steep and persistent thermal gradient, with the interior of the brick remaining significantly cold throughout the day. The PCM in this scenario remains permanently frozen and inert. In stark contrast, the heated brick (right column) demonstrates the formation of a warm thermal core. During the charging phase (top right), the heat from the electrical element is absorbed, raising the temperature of the PCM core above its 10 °C melting point, as indicated by the yellow and red regions. This is the stored latent and sensible heat. This thermal storage can be quantified using the temperature data from the “peak charge” phase in Fig. 6 (right column, second from top) and the properties in Table 1. In this state, the PCM core reaches temperatures of approximately 18 °C. The total energy stored per kilogram of Pentadecane can be broken down into three stages. First, initial sensible heat is absorbed as the solid PCM heats from the sub-zero ambient conditions to its melting range around 10 °C. Second, a substantial amount of latent heat—208,000 J/kg—is absorbed during the phase change. Finally, additional sensible heat is stored as the liquid PCM is heated from approximately 10 °C to a peak of 18 °C. Based on a specific heat capacity of 2070 J/kg K, this final sensible heat gain alone accounts for over 16,500 J/kg (2070 J/kg K × 8 K). This quantification clearly shows that the latent heat component is overwhelmingly dominant, representing the bulk of the brick’s “thermal battery” capacity and confirming the critical role of the phase change process in the system’s effectiveness.

During the discharge phase (bottom right), this warm core persists even as the outer layers of the brick cool down. This stored energy creates a thermal gradient that drives heat flow both inward toward the room and outward toward the freezing exterior. The relatively uniform temperature distribution within the PCM enclosure is direct visual evidence of the Copper Oxide Foam’s critical role. Without this high-conductivity foam matrix, the poor thermal conductivity of the PCM would lead to significant temperature gradients, localized overheating near the heating element, and incomplete melting of the PCM volume. The COF effectively creates a thermal scaffold, ensuring that heat is distributed rapidly and evenly, which is essential for both efficient charging and preventing material degradation. These visualizations confirm that the active system successfully creates a high-inertia thermal core, enabling the brick to function as a controllable thermal battery.

## Conclusion

This study numerically investigated a novel brick integrated with a Phase Change Material and an electrical heating element, designed to function as an active thermal battery for buildings in freezing climates. The research successfully demonstrated that the proposed system can transform a thermally inefficient building envelope into a valuable energy storage and radiant heating component. The findings confirm that without intervention, the brick’s indoor surface can fall to temperatures as low as − 5 °C, creating significant thermal discomfort and a high heating load. By activating the integrated heater, the system fundamentally changes the wall’s role from a thermal liability to an active thermal barrier. It not only prevents heat loss but also provides supplemental heat to the indoor space. By maintaining the indoor surface temperature above 8 °C, even when the ambient temperature is − 20 °C, the system significantly improves the indoor radiant environment by eliminating an extremely cold surface and directly reduces the load on the primary HVAC system.

Quantitatively, the system’s ability to shift electrical loads was profound. By storing off-peak energy, the active brick delivered a peak heat output of over 150 W/m^2^ during critical daytime hours, directly offsetting the demand on the primary HVAC system. his resulted in a reduction of the wall’s net daily energy loss by nearly 70%. Qualitatively, the visualizations confirmed that the integrated high-conductivity Copper Oxide Foam was essential to the system’s success, ensuring uniform heat distribution for efficient charging and facilitating a stable heat discharge by overcoming the inherent low thermal conductivity of the PCM. The latent heat capacity of the PCM was crucial in stabilizing the heat discharge, providing a more consistent and comfortable radiant warmth compared to a simple heating element alone. In conclusion, this active brick system presents a highly effective and viable solution for enhancing energy resilience, improving occupant comfort, and significantly reducing peak heating demand in cold regions.

For future work, the most critical next step is the experimental validation of these numerical results through the fabrication and testing of a physical prototype. Further research should also focus on optimizing the charging and discharging cycles through advanced control strategies and integrating the model into whole-building energy simulations to assess its annual performance and grid-level impact.

## Data Availability

The datasets used and/or analyzed during the current study are available from the corresponding author upon reasonable request.

## Abbreviations

CFD: Computational fluid dynamics
COF: Copper oxide foam
FVM: Finite volume method
PCM: Phase change material
PCM-B: Phase change material-brick
Pdc: Daily peak demand contribution
TPP: Thermophysical properties

## List of symbols

A: Area (m^2^)
C_p_: Specific heat capacity at constant pressure (J/kg K)
d_p_: Pore diameter of foam (m)
g: Gravitational acceleration vector (m/s^2^)
h: Convective heat transfer coefficient (W/m^2^ K)
h: Latent heat of fusion (J/kg)
k: Thermal conductivity (W/m K)
K: Permeability of porous medium (mÂ^2^)
P: Pressure (Pa)
p: Integrated heating power per unit area
q′′: Heat flux (W/mÂ^2^)
S: Momentum sink term (kg/m^2^ s)
t: Time (s)
T: Temperature (K or °C)
V: Velocity vector (m/s)

## Greek symbols

$$\alpha$$: Solar absorptivity (–)
$\beta$: Thermal expansion coefficient (1/K)
$\Delta$: Difference or change (–)
$\varepsilon$: Porosity of foam (–)
Ɛ: Surface emissivity (–)
$$\lambda$$: Liquid fraction (–)
$\mu$: Dynamic viscosity (Pa s)
$$\rho$$: Density (kg/m^3^)
$\sigma$: Stefan-Boltzmann constant ($5.6 \times 10^{ - 8} \,{\text{W}}/{\text{m}}^{2} \;{\text{K}}^{{4}}$)

## Subscripts

af: Stainless steel foam
b: Brick
d: Daily
cof: Copper oxide foam
i: Indoor
is: Indoor surface
m: Melting
max: Maximum
min: Minimum
mr: Melting range
o: Outdoor
os: Outdoor surface
pcm: Phase change material
sky: Sky
sun: Solar
